# The stochastic Fitzhugh–Nagumo neuron model in the excitable regime embeds a leaky integrate-and-fire model

**DOI:** 10.1007/s00285-019-01366-z

**Published:** 2019-05-02

**Authors:** Marius E. Yamakou, Tat Dat Tran, Luu Hoang Duc, Jürgen Jost

**Affiliations:** 1 Max Planck Institute for Mathematics in the Sciences, Inselstraße 22, 04103 Leipzig, Germany; 2 Institute of Mathematics, Vietnam Academy of Science and Technology, 18 Hoang Quoc Viet Road, 10307 Ha Noi, Vietnam; 3 Santa Fe Institute for the Sciences of Complexity, Santa Fe, NM 87501 USA

**Keywords:** FitzHugh–Nagumo model, Excitable regime, Leaky integrate-and-fire model, Random attractor, Stationary distribution, 60GXX, 92BXX

## Abstract

In this paper, we provide a complete mathematical construction for a stochastic leaky-integrate-and-fire model (LIF) mimicking the interspike interval (ISI) statistics of a stochastic FitzHugh–Nagumo neuron model (FHN) in the excitable regime, where the unique fixed point is stable. Under specific types of noises, we prove that there exists a global random attractor for the stochastic FHN system. The linearization method is then applied to estimate the firing time and to derive the associated radial equation representing a LIF equation. This result confirms the previous prediction in Ditlevsen and Greenwood (J Math Biol 67(2):239–259, 2013) for the Morris-Lecar neuron model in the bistability regime consisting of a stable fixed point and a stable limit cycle.

## Introduction

Mathematical modeling has emerged as an important tool to handle the overwhelming structural complexity of neuronal processes and to gain a better understanding of their functioning from the dynamics of their model equations. However, the mathematical analysis of biophysically realistic neuron models such as the 4-dimensional Hodgkin–Huxley (HH) (1952) and the 2-dimensional Morris–Lecar (ML) (1981) equations is difficult, as a result of a large parameter space, strong nonlinearities, and a high dimensional phase space of the model equations. The search for simpler, mathematically tractable (small parameter space, weaker nonlinearities, low dimensional phase space) neuron models that still capture all, or at least some important dynamical behaviors of biophysical neurons (HH and ML) has been an active area of research.

The efforts in this area of research have resulted in easily computable neuron models which mimic some of the dynamics of biophysical neuron models. One of the resulting models is the 2-dimensional FitzHugh–Nagumo (FHN) neuron model (FitzHugh 1961). The FHN model has been so successful, because it is at the same time mathematically simple and produces a rich dynamical behavior that makes it a model system in many regards, as it reproduces the main dynamical features of the HH model. In fact, the HH model has two types of variables, and each type then is combined into a single variable in FHN: The (*V*, *m*) variables of HH correspond to the *v* variable in FHN, whose fast dynamics represents excitability; the (*h*, *n*) variables correspond to the *w* variable, whose slow dynamics represents accommodation and refractoriness.

The fact that the FHN model is low dimensional makes it possible to visualize the solution and to explain in geometric terms important phenomena related to the excitability and action potential generation mechanisms observed in biological neurons. Of course, this comes at the expense of numerical agreement with the biophysical neuron models (Yamakou 2018). The purpose of the model is not a close match with biophysically realistic high dimensional models, but rather a mathematical explanation of the essential dynamical mechanism behind the firing of a neuron. Moreover, the analysis of such simpler neuron models may lead to the discovery of new phenomena, for which we may then search in the biological neuron models and also in experimental preparations.

There is, however, an even simpler model than FHN, the leaky integrate-and-fire model (LIF). This is the simplest reasonable neuron model. It only requires a few basic facts about nerve cells: they have membranes, they are semipermeable, and they are polarizable. This suffices to deduce a circuit equivalent to that of the membrane potential of the neuron: a resistor-capacitor circuit. Such circuits charge up slowly when presented with a current, cross a threshold voltage (a spike), then slowly discharge. This behavior is modeled by a simple 1D equation together with a reset mechanism: the leaky integrate-and-fire neuron model equation (Gerstner and Kistler 2002). Combining sub-threshold dynamics with firing rules has led to a variety of 1D leaky integrate-and-fire descriptions of a neuron with a fixed membrane potential firing threshold (Gerstner and Kistler 2002; Lansky and Ditlevsen 2008) or with a firing rate depending more sensitively on the membrane potential (Pfister et al. 2006). In contrast to $n-$dimensional neuron models, $n\ge 2$, such as the HH, ML, and FHN models, the LIF class of neuron models is less expensive in numerical simulations, which is an essential advantage when a large network of coupled neurons is considered.

Noise is ubiquitous in neural systems and it may arise from many different sources. One source may come from synaptic noise, that is, the quasi-random release of neurotransmitters by synapses or random synaptic input from other neurons. As a consequence of synaptic coupling, real neurons operate in the presence of synaptic noise. Therefore, most works in computational neuroscience address modifications in neural activity arising from synaptic noise. Its significance can however be judged only if its consequences can be separated from the internal noise, generated by the operations of ionic channels (Calvin and Stevens 1967). The latter is channel noise, that is, the random switching of ion channels. In many papers channel noise is assumed to be minimal, because typically a large number of ion channels is involved and fluctuations should average out, and therefore, the effects of synaptic noise should dominate. Consequently, channel noise is frequently ignored in the mathematical modeling. However, the presence of channel noise can also greatly modify the behavior of neurons (White et al. 2000). Therefore, in this paper, we study the effect of channel noise. Specifically, we add a noise term to the right-hand side of the gating equations (the equation for the ionic current variable).

In the stochastic model, the deterministic fixed point is no longer a solution of the system. The fixed point necessarily needs to vary and adapt to the noise. To account for this, in the theory of random dynamical systems, the notion of a random dynamical attractor was developed as a substitute for deterministic attractors in the presence of noise. In the first part of this paper, we therefore prove that our system admits a global random attractor, for both additive and multiplicative channel noises. This can be seen as a theoretical grounding of our setting.

In Ditlevsen and Greenwood (2013), it was shown that a stochastic LIF model constructed with a radial Ornstein–Uhlenbeck process is embedded in the ML model (in a bistable regime consisting of a fixed point and limit cycle) as an integral part of it, closely approximating the sub-threshold fluctuations of the ML dynamics. This result suggests that the firing pattern of a stochastic ML can be recreated using the embedded LIF together with a ML stochastic firing mechanism. The LIF model embedded in the ML model captures sub-threshold dynamics of a combination of the membrane potential and ion channels. Therefore, results that can be readily obtained for LIF models can also yield insight about ML models. In the second part of this paper, we here address the problem to obtain a stochastic LIF model mimicking the interspike interval (ISI) statistics of the stochastic FHN model in the excitable regime, where the unique fixed point is stable. Theoretically, we obtain such a LIF model by reducing the 2D FHN model to the one dimensional system that models the distance of the solution to the random attractor as shown in the first part of the paper. In fact, we show that this distance can be approximated to the fixed point, up to a rescaling, as the Euclidean norm $R_t$ of the solution of the linearization of the stochastic FHN equation along the deterministic equilibrium point, and hence the LIF model is approximated by the equation for $R_t$. An action potential (a spike) is produced when $R_t$ exceeds a certain firing threshold $R_t\ge r_0>0$. After firing the process is reset and time is back to zero. The ISI $\tau _0$ is identified with the first-passage time of the threshold, $\tau _0=\inf \{t>0: R_t\ge r_0>0\}$, which then acts as an upper bound of the spiking time $\tau$ of the original system. By defining the firing as a series of first-passage times, the 1D radial process $R_t$ together with a simple firing mechanism based on the detailed FHN model (in the excitable regime), the firing statistics is shown to reproduce the 2D FHN ISI distribution. We also show that $\tau$ and $\tau _0$ share the same distribution.

The rest of the paper is organized as follows: Sect. 2 introduces the deterministic version of the FHN neuron model, where we determine the parameter values for which the model is in the excitable regime. In Sect. 3, we prove the existence of a global random attractor of the random dynamical system generated by the stochastic FHN equation; and furthermore derive a rough estimate for the firing time using the linearization method. The corresponding stochastic LIF equation is then derived in Sect. 4 and its distribution of interspike-intervals is found to numerically match the stochastic FHN model.

## The deterministic model and the excitable regime

In the fast time scale *t*, the deterministic FHN neuron model is

2.1 $$\begin{aligned} {\left\{ \begin{array}{ll} dv_t= \left( v_t-\displaystyle {\frac{v_t^3}{3}}-w_{t}+I\right) dt =f(v_t,w_t)dt,\\ dw_t= \varepsilon (v_t+\alpha -\beta w_t)dt=g(v_t,w_t)dt, \end{array}\right. } \end{aligned}$$

where $v_t$ is the activity of the membrane potential and $w_t$ is the recovery current that restores the resting state of the model. *I* is a constant bias current which can be considered as the effective external input current. $0<\varepsilon :=t/\tau \ll 1$ is a small singular perturbation parameter which determines the time scale separation between the fast *t* and the slow time scale $\tau$. Thus, the dynamics of $v_t$ is much faster than that of $w_t$. $\alpha$ and $\beta$ are parameters.

The deterministic critical manifold $\mathcal {C}_0$ defining the set of equilibria of the *layer problem* associated to Eq. (2.1) (i.e., the equation obtained from Eq. (2.1) in the singular limit $\epsilon = 0$, see Kuehn (2015) for a comprehensive introduction to slow-fast analysis), is obtained by solving $f(v,w)=0$ for *w*. Thus, it is given by

2.2 $$\begin{aligned} \mathcal {C}_0=\left\{ (v,w)\in \mathbb {R}^2: w=\displaystyle {v-\frac{v^3}{3}+I}\right\} . \end{aligned}$$

**Fig. 1:**
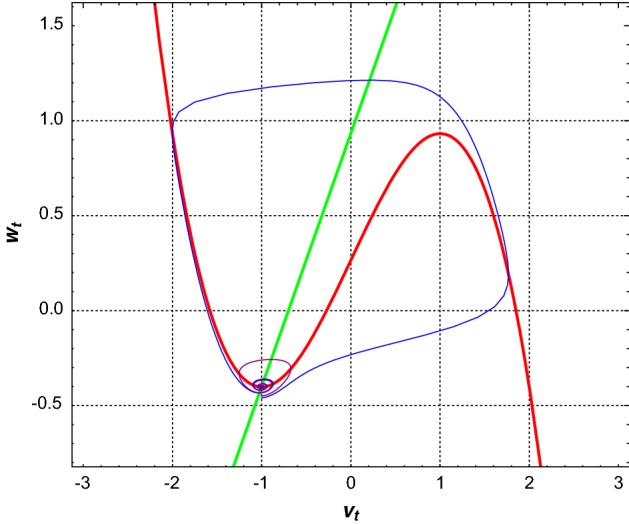
The critical manifold $\mathcal {C}_0$ (red curve) and the *w*-nullcline (green line) intersect at the unique and stable fixed point $(v_e,w_e)=(-1.00125,-0.401665)$. Two deterministic trajectories are shown, the purple curve starts at $(-1.00125, -0.45)$ and the blue curve starts at $(-1.00125, -0.46)$. Parameters of the system $I = 0.265, \alpha =0.7, \beta =0.75, \varepsilon =0.08$ and the real time for trajectories $T=1000$ (color figure online)

We note that for Eq. (2.1), $\mathcal {C}_0$ coincides with the *v*-nullcline (the red curve in Fig. (1)). The stability of points on $\mathcal {C}_0$ as steady states of the *layer problem* associated to Eq. (2.1) is determined by the Jacobian scalar $(D_vf)(v,w)=1-v^2$. This shows that on the critical manifold, points with $|v|>1$ are stable while points with $|v|<1$ are unstable. It follows that the branch $v_{-}^*(w)\in (-\infty ,-1)$ is stable, $v_0^*(w)\in (-1,1)$ is unstable, and $v_+^*(w)\in (1,+\infty )$ is stable.

The set of fixed points $(v_e,w_e)$ which define the resting states of the neuron is given by

2.3 $$\begin{aligned} \{(v,w)\in \mathbb {R}^2:f(v,w)=g(v,w)=0\}. \end{aligned}$$

The sign of the discriminant $\bigtriangleup = (1/\beta -1)^3+\frac{9}{4}(\alpha /\beta -I)^2$, determines the number of fixed points. $\mathcal {C}_0$ can therefore intersect the *w*-nullcline ($w=\frac{v+\alpha }{\beta }$) at one, two or three different fixed points. We assume in this paper that $\bigtriangleup >0$, in which case we have a unique fixed point given by

2.4 $$\begin{aligned} {\left\{ \begin{array}{ll} \displaystyle {v_{e}=\root 3 \of {-\frac{q}{2}-\sqrt{\Delta }}+\root 3 \of {-\frac{q}{2}+\sqrt{\Delta }}}\\ w_{e}=\frac{1}{\beta }(v_{e}+\alpha ). \end{array}\right. } \end{aligned}$$

where

$$\begin{aligned} p=3\Big (\frac{1}{\beta }-1\Big ), \qquad q=3\Big (\frac{\alpha }{\beta } - I\Big ). \end{aligned}$$

Here, we want to consider the neuron in the excitable regime (Ditlevsen and Greenwood 2013). A neuron is in the excitable regime when starting in the basin of attraction of a unique stable fixed point, an external pulse will result into at most one large excursion (spike) into the phase space after which the phase trajectory returns back to this fixed point and stays there (Izhikevich 2007).

In order to have Eq. (2.1) in the excitable regime, we choose $I, \alpha ,$ and $\beta$ such that $\Delta >0$ (i.e., a unique fixed point) and $\varepsilon$ such that the Jacobian (the linearization matrix *M*) of Eq. (2.1) at the fixed point $(v_e,w_e)$ has a pair of complex conjugate eigenvalues

$$\begin{aligned} -\mu \pm i \nu = \frac{1}{2}\left( 1-v_e^2 - \epsilon \beta \right) \pm \frac{i}{2} \sqrt{4\epsilon - \left( 1-v_e^2+\epsilon \beta \right) ^2} \end{aligned}$$

with negative real part (i.e., a stable fixed point). In that case, $(v_e,w_e)$ is the only stationary state and there is no limit cycle of system (2.1). In other words, $(v_e,w_e)$ is the global attractor of the system (Izhikevich 2007). Moreover, to apply the averaging technique (Baxendale and Greenwood 2011), it is necessary that $\mu \ll \nu$, we therefore use throughout this paper the following parameters of the system: $I = 0.265, \alpha =0.7, \beta =0.75, \varepsilon =0.08$ so that $(v_e,w_e) = (-1.00125,-0.401665)$ is the unique stable fixed point and $\frac{\mu }{\nu } = 0.111059 \ll 1$. Figure (1) shows the neuron in the excitable regime. Notice that although every trajectory finally converges to the fixed point, only a small change in the location of the starting point will result in different behavior of the trajectories (see the blue and purple curves).

## The stochastic model

We consider this stochastic FHN model

3.1 $$\begin{aligned} {\left\{ \begin{array}{ll} dv_t= f(v_t,w_t)dt, \\ dw_t= g(v_t,w_t)dt+h(w_t)\circ dB_t, \end{array}\right. } \end{aligned}$$

where the deterministic fields *f* and *g* are given in Eq. (2.1). There are two important cases: either $h(w) = \sigma _0$ (additive channel noise) or $h(w)=\sigma _0 w$ (multiplicative channel noise). $\circ dB_t$ stands for the Stratonovich stochastic integral with respect to the Brownian motion $B_t$.

**Fig. 2:**
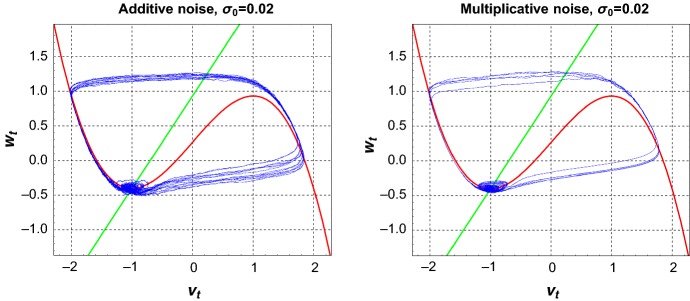
Random trajectory of Eq. (3.1) in the excitable regime with chosen parameters of the system and the initial condition $(v_0,w_0) = (-1.00125,-0.4)$ for both additive and multiplicative noise (we use the StochasticRungeKutta method in *Mathematica* with the real time $T=1000$ and the step size $h=0.01$)

Figure 2 shows the phase portraits of Eq. (3.1) starting with the initial condition $(v_0,w_0) = (-1.00125,-0.4)$, which is in the vicinity of the stable fixed point. Given an initial condition close to the stable fixed point $(v_e,w_e)=(-1.00125,-0.401665)$, the trajectory of the stochastic system might first rotate around the stable fixed point but then the noise may trigger a spike, that is, a large excursion into the phase space, before returning to the neighbourhood of the fixed point; the process repeats itself leading to alternations of small and large oscillations. A similar behavior can be observed when the deterministic system with an additional limit cycle is perturbed by noise (as seen in the bistable system Ditlevsen and Greenwood 2013).

**Fig. 3:**
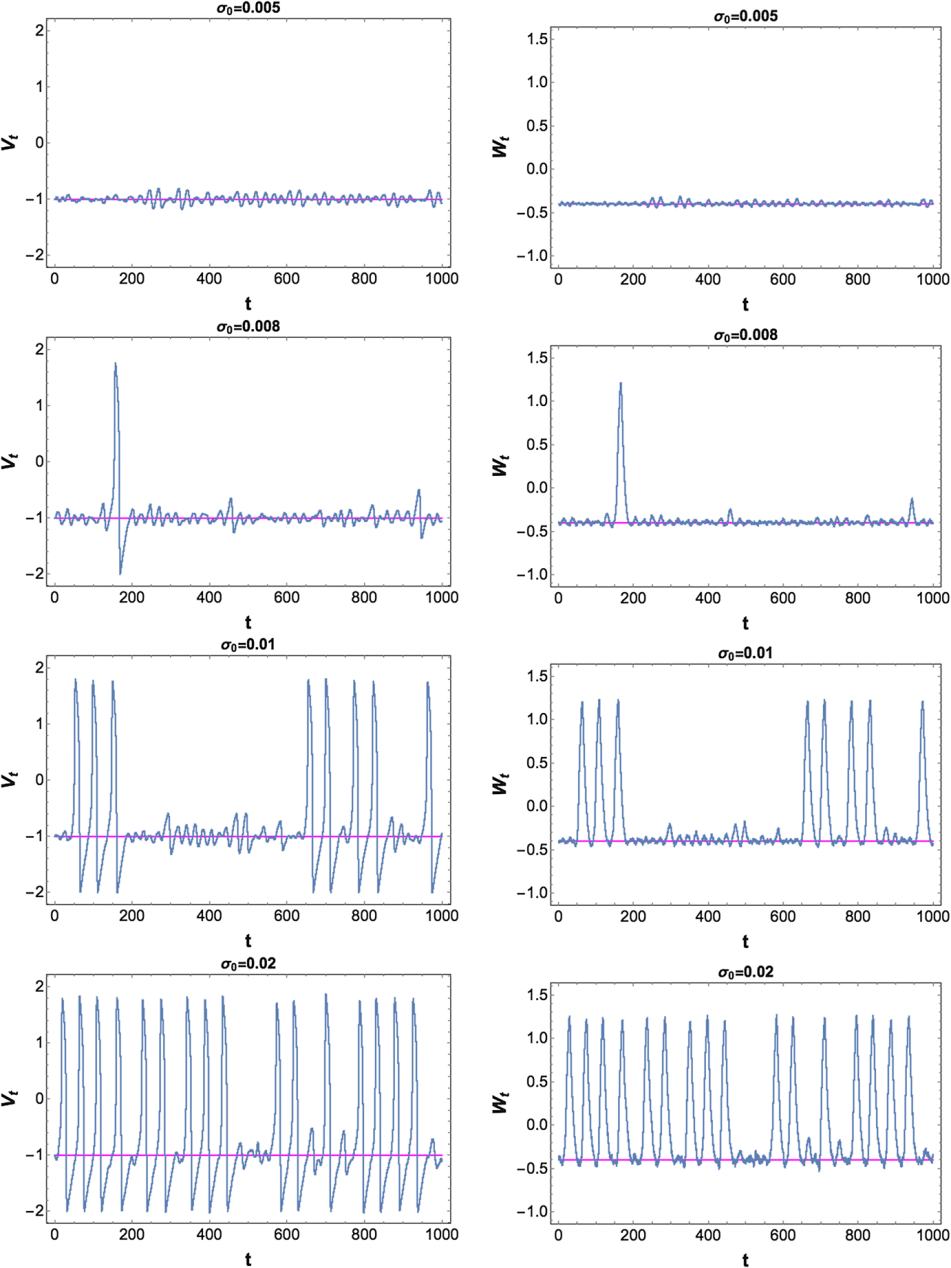
The components (left column: $V_t$, right column: $W_t$) of a random trajectory of Eq. (3.1) in the excitable regime with chosen parameters of the system and the initial condition $(v_0,w_0) = (-1.00125,-0.4)$ for additive noise with $\sigma _0 \in \{0.005, 0.008, 0.01, 0.02\}, T=1000, h=0.01$

Figure 3 shows that the spiking frequency increases as the amplitude of the noise increases. For a fixed simulation time $T=1000$, the system spikes only rarely, if at all, when the amplitude $\sigma _0 \le 0.005$, but spikes more frequently when $\sigma _0$ increases. This is similar for multiplicative noise.

Let $\mathbf X= (v,w)^T$ and $F(\mathbf X),H(\mathbf X) \in \mathbb {R}^2$ be the drift and diffusion coefficients of (3.1). The stochastic system is then of the form

3.2 $$\begin{aligned} d \mathbf X_t = F(\mathbf X_t)dt + H(\mathbf X_t) \circ dB_t, \end{aligned}$$

where $H(\mathbf X) = (0,\sigma _0)^\mathrm{T}$ for additive noise and $$ H(\mathbf X) = \begin{pmatrix}0&{}0\\ 0&{}\sigma _0 \end{pmatrix} \mathbf X=B\mathbf X$$ for multiplicative noise. It is easy to check that *F* is dissipative in the weak sense, i.e.

3.3 $$\begin{aligned}&\langle \mathbf X_1 - \mathbf X_2,F(\mathbf X_1)-F(\mathbf X_2) \rangle \nonumber \\&\quad = (v_1-v_2)^2 \Big [1- \frac{1}{3} \left( v_1^2 + v_1 v_2 + v_2^2\right) \Big ]\nonumber \\&\quad \quad -(1-\epsilon )(v_1-v_2)(w_1-w_2) - \epsilon \beta (w_1-w_2)^2\nonumber \\&\quad \le (v_1-v_2)^2 \Big [1 - \frac{1}{12} (v_1-v_2)^2\Big ]\nonumber \\&\quad \quad + \frac{(1-\epsilon )^2}{2 \epsilon \beta } |v_1-v_2|^2 + \frac{\epsilon \beta }{2}|w_1-w_2|^2 - \epsilon \beta (w_1-w_2)^2\nonumber \\&\quad \le -\frac{1}{12} \Bigg ( |v_1-v_2|^2 - 6\left( 1+ \frac{\epsilon \beta }{2} + \frac{(1-\epsilon )^2}{2 \epsilon \beta }\right) \Bigg )^2 \end{aligned}$$

3.4 $$\begin{aligned}&\quad \quad +3\left( 1+ \frac{\epsilon \beta }{2} + \frac{(1-\epsilon )^2}{2 \epsilon \beta }\right) ^2- \frac{\epsilon \beta }{2} (|v_1-v_2|^2+|w_1-w_2|^2) \nonumber \\&\quad \le a- b \Vert \mathbf X_1 - \mathbf X_2\Vert ^2 \end{aligned}$$

where

$$\begin{aligned} a := 3\left( 1+ \frac{\epsilon \beta }{2} + \frac{(1-\epsilon )^2}{2 \epsilon \beta }\right) ^2, \qquad b:= \frac{\epsilon \beta }{2}. \end{aligned}$$

On the other hand, we have

3.5 $$\begin{aligned} |H(\mathbf X_1)-H(\mathbf X_2)| \le \sigma _0 \Big | w_1-w_2 \Big | \le \sigma _0 \Vert \mathbf X_1-\mathbf X_2\Vert , \end{aligned}$$

for multiplicative noise, while $|H(\mathbf X_1)-H(\mathbf X_2)| \equiv 0$ for additive noise, so *H* is globally Lipschitz continuous.

### The existence of a random attractor

In the sequel, we are going to prove that there exists a unique solution $\mathbf X(\cdot ,\omega ,\mathbf X_0)$ of (3.1) and the solution then generates a so-called *random dynamical system* (see e.g. Arnold 1998, Chapters 1–2).

More precisely, let $(\Omega ,\mathcal {F},\mathbb {P})$ be a probability space on which our Brownian motion $B_t$ is defined. In our setting, $\Omega$ can be chosen as $C^0(\mathbb {R},\mathbb {R})$, the space of continuous real functions on $\mathbb {R}$ which are zero at zero, equipped with the compact open topology given by the uniform convergence on compact intervals in $\mathbb {R}$, $\mathcal {F}$ as $\mathcal {B}(C^0)$, the associated Borel-$\sigma$-algebra and $\mathbb {P}$ as the Wiener measure. The Brownian motion $B_t$ can then be constructed as the canonical version $B_t(\omega ) := \omega (t)$.

On this probability space we construct a dynamical system $\theta$ as the Wiener shift

3.6 $$\begin{aligned} \theta _t m(\cdot )=m(t+\cdot )-m(t),\quad \forall t\in \mathbb {R}, \forall m \in \bar{\Omega }. \end{aligned}$$

Then $\theta _t(\cdot ): \Omega \rightarrow \Omega$ satisfies the group property, i.e. $\theta _{t+s} = \theta _t \circ \theta _s$ for all $t,s \in \mathbb {R}$, and is $\mathbb {P}$-preserving, i.e. $\mathbb {P}(\theta _t^{-1}(A)) = \mathbb {P}(A)$ for every $A \in \mathcal {F}$, $t \in \mathbb {R}$. The quadruple $((\Omega ,\mathcal {F},\mathbb {P},(\theta _t)_{t\in \mathbb {R}})$ is called a *metric dynamical system*.

Given such a probabilistic setting, Theorem 3.1 below proves that the solution mapping $\varphi : \mathbb {R}\times \Omega \times \mathbb {R}^2 \rightarrow \mathbb {R}^2$ defined by $\varphi (t,\omega )\mathbf X_0 := \mathbf X(t,\omega ,\mathbf X_0)$ is a random dynamical system satisfying $\varphi (0,\omega )\mathbf X_0 = \mathbf X_0$ and the cocycle property

3.7 $$\begin{aligned} \varphi (t+s,\omega )\mathbf X_0 = \varphi (t,\theta _s \omega )\circ \varphi (s,\omega )\mathbf X_0,\qquad \forall t,s \in \mathbb {R}, \omega \in \Omega , \mathbf X_0 \in \mathbb {R}^2 \end{aligned}$$

To investigate the asymptotic behavior of the system under the influence of noise, we shall first check the effect of the noise amplitude on firing. Under the stochastic scenario, the fixed point $\mathbf X_e=(v_e,w_e)$ is no longer the stationary state of the stochastic system (3.1). Instead, we need to find the global asymptotic state as a compact random set $A(\omega ) \in \mathbb {R}^2$ depending measurably on $\omega \in \Omega$ such that *A* is invariant under $\varphi$, i.e. $\varphi (t,\omega )A(\omega ) = A(\theta _t \omega )$, and attracts all other compact random sets $D(\omega )$ in the pullback sense, i.e.

$$\begin{aligned} \lim \limits _{t\rightarrow \infty } d(\varphi (t,\theta _{-t}\omega )D(\theta _{-t}\omega ) | A(\omega )) = 0, \end{aligned}$$

where *d*(*B*|*A*) is the Hausdorff semi-distance. Such a structure is called a *random attractor* (see e.g. Crauel et al. 1997 or Arnold 1998, Chapter 9).

The following theorem ensures that the stochastic system (3.1) has a global random pullback attractor. The proof is provided in the “Appendix”.

#### Theorem 3.1

There exists a unique solution of (3.2) which generates a random dynamical system. Moreover, the system possesses a global random pullback attractor.

Theorem 3.1 shows that every trajectory would in the long run converge to the global random attractor. The structure and the inside dynamics of the global random attractor are still open issues which might help understand the firing mechanism.

### The normal form at the equilibrium point

One way to study the dynamics of the stochastic system (3.1) is through its linearization. Therefore, in this section, we shall study the dynamics of (3.1) in a small vicinity of the fixed point $\mathbf X_e=(v_e,w_e)$. To do that, consider the shift system w.r.t. the fixed point $\mathbf X_e$ which has the form

3.8 $$\begin{aligned} d(\mathbf X_t -\mathbf X_e)= & {} [F(\mathbf X_t) - F(\mathbf X_e)] dt + H(\mathbf X_t) \circ dB_t \nonumber \\= & {} \Big [DF(\mathbf X_e) (\mathbf X_t-\mathbf X_e) + \bar{F}(\mathbf X_t-\mathbf X_e)\Big ] dt + H(\mathbf X_t) \circ dB_t, \end{aligned}$$

with initial point $\mathbf X_0-\mathbf X_e$, where $DF(\mathbf X_e)$ is the linearized matrix of *F* at $\mathbf X_e$, $\bar{F}$ is the nonlinear term such that

$$\begin{aligned} \begin{aligned} \Vert \bar{F}(\mathbf X-\mathbf X_e)\Vert&= \Bigg \Vert \begin{pmatrix} \frac{1}{3}|v+2v_e|(v-v_e)^2 \\ 0 \end{pmatrix}\Bigg \Vert \\&\le \gamma (r) \Vert \mathbf X-\mathbf X_e\Vert ,\qquad \forall \Vert \mathbf X-\mathbf X_e\Vert \le r \end{aligned} \end{aligned}$$

for an increasing function $\gamma (\cdot ): \mathbb {R}_+\rightarrow \mathbb {R}_+$, $r\mapsto \frac{r^2}{3} + |v_e|r$, which implies that $\lim \limits _{r \rightarrow 0} \gamma (r) =0$. Since $H(\mathbf X)$ is either a constant or a linear function, we prove below that system (3.8) can be well approximated by its linearized system

3.9 $$\begin{aligned} d{\bar{\mathbf X}}_t = DF(\mathbf X_e) {\bar{\mathbf X}}_t dt + H(\bar{\mathbf X}_t + \mathbf X_e) \circ dB_t,\qquad \bar{\mathbf X}_0 = \mathbf X_0 -\mathbf X_e. \end{aligned}$$

#### Theorem 3.2

Given $\Vert \mathbf X_0-\mathbf X_e\Vert < r$ and equations (3.8), (3.9), define the stopping time $\tau = \inf \{t>0: \Vert \mathbf X_t - \mathbf X_e\Vert \ge r\}$. Then there exists a constant *C* independent of *r* such that for any $t\ge 0$, the following estimates hold

- For additive noise
  3.10 $$\begin{aligned} \sup _{t\le \tau }\Vert \mathbf X_t - \mathbf X_e -\bar{\mathbf X}_t\Vert \le C \gamma (r) r. \end{aligned}$$
- For multiplicative noise
  3.11 $$\begin{aligned} E\Vert \mathbf X_{t\wedge \tau } - \mathbf X_e -\bar{\mathbf X}_{t\wedge \tau }\Vert ^2 \le C \gamma ^2(r) r^2. \end{aligned}$$

The proof is provided in the “Appendix”. In practice we can even approximate (3.8) by the following linear system with additive noise

3.12 $$\begin{aligned} d{\tilde{\mathbf X}}_t = DF(\mathbf X_e) \tilde{\mathbf X}_t dt + H(\mathbf X_e) \circ dB_t,\qquad \tilde{\mathbf X}_0 = \mathbf X_0 -\mathbf X_e. \end{aligned}$$

By the same arguments as in the proof of Theorem 3.2, we can prove the following estimate

3.13 $$\begin{aligned} E\Vert \mathbf X_{t\wedge \tau } - \mathbf X_e -\tilde{\mathbf X}_{t\wedge \tau }\Vert ^2 \le C r_0^2, \end{aligned}$$

for the same stopping time $\tau = \inf \{t>0: \Vert \mathbf X_t - \mathbf X_e\Vert \ge r_0\}$.

**Fig. 4:**
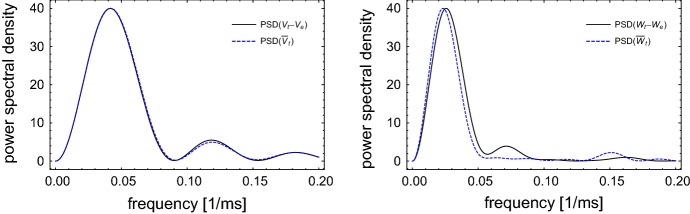
The process $\{\mathbf X_t -\mathbf X_e\}_t$ (3.8) and the process $\{\bar{\mathbf X}_t\}_t$ (3.9) with the chosen parameters of the system, $\sigma _0=0.01$ and the same starting point $(v_0-v_e,w_0-w_e)$ are compared by using the power spectral density. Their spectrum densities are well approximated

Another comparison between the processes $\{\mathbf X_t -\mathbf X_e\}_t$ and $\{\bar{\mathbf X}_t\}_t$ can be obtained by using power spectral density estimation (see, for example, Fan and Yao 2003, Chapter 7). In Fig. 4, the estimated spectral densities of the shifted original and the linearized process are plotted. The spectral densities are estimated from paths started from 0 to 50 ms of subthreshold fluctuations, and scaled to have the same maximum at 40.

## The embedded LIF model

In this section, we present two constructive methods to obtain 1-D LIF models corresponding to the stochastic FHN in the excitable regime in Eq. (3.1). The first method follows Baxendale and Greenwood (2011) (see also Ditlevsen and Greenwood 2013) by constructing the so-called *radial Ornstein–Uhlenbeck equation*. More precisely, we rewrite the linearized system (3.9) in the form

4.1 $$\begin{aligned} d\bar{\mathbf X}_t = M \bar{\mathbf X}_t dt + \begin{pmatrix}0 &{}\, 0\\ 0 &{}\, \sigma _0\end{pmatrix} d\mathbf B_t, \end{aligned}$$

where $M= DF(\mathbf X_e)$ and $$\mathbf B_t = \begin{pmatrix}B'_t\\ B_t\end{pmatrix}$$ is a 2-D standard Brownian motion. For chosen parameters, *M* has a pair of complex conjugate eigenvalues $-\mu \pm i \nu$ with $\mu = 0.0312496, \nu = 0.281378$. By transformation $\bar{\mathbf Y}_t = Q^{-1} \bar{\mathbf X}_t$ with $$Q = \begin{pmatrix}-\nu &{}\, m_{11}+\mu \\ 0 &{}\, m_{21}\end{pmatrix}$$ we obtain

4.2 $$\begin{aligned} d \bar{\mathbf Y}_t = A\bar{\mathbf Y}_t dt + C d\mathbf B_t, \end{aligned}$$

where

$$\begin{aligned} A= & {} \begin{pmatrix}-\mu &{}\, \nu \\ -\nu &{}\, -\mu \end{pmatrix} = \begin{pmatrix}-0.0312496 &{}\, 0.281378\\ -0.281378 &{}\, -0.0312496\end{pmatrix} ; \\ C= & {} Q^{-1} \begin{pmatrix}0 &{}\, 0\\ 0 &{}\, \sigma _0\end{pmatrix}. \end{aligned}$$

We note that $\frac{\mu }{\nu } = 0.111059 \ll 1$, therefore, by applying the technique of time average from (Baxendale and Greenwood 2011, Theorem 1), $\bar{\mathbf Y}_t$ can be approximated by an Ornstein-Uhlenbeck process up to a rotation, i.e.

$$\begin{aligned} \bar{\mathbf Y}_t \sim \bar{\mathbf Y}^{app}_t := \frac{\sigma }{\sqrt{\mu }} Rot_{-\nu t} \bar{\mathbf S}_{\mu t}, \end{aligned}$$

where $\sigma = \sqrt{\frac{1}{2} \mathrm {tr} (C C^*)} = \sqrt{\frac{-m_{12}}{2 \nu ^2 m_{21}}}\sigma _0$, the rotation

$$\begin{aligned} Rot_s := \begin{pmatrix}\cos s &{}\, -\sin s\\ \sin s&{}\, \cos s\end{pmatrix}, \end{aligned}$$

and $\bar{\mathbf S}_t$ is the unique solution of the 2-D SDE

$$\begin{aligned} d\bar{\mathbf S}_t = -\bar{\mathbf S}_t dt + d \mathbf B_t, \end{aligned}$$

with the initial value $\bar{\mathbf S}_0=\frac{\sqrt{\mu }}{\sigma }\bar{\mathbf Y}_0$. Therefore, $\Vert \bar{\mathbf Y}_t\Vert$ can be approximated by $R_t := \Vert \bar{\mathbf Y}^{app}_t\Vert = \frac{\sigma }{\sqrt{\mu }}\Vert \bar{\mathbf S}_{\mu t}\Vert$ which by Ito calculus satisfies the SDE

4.3 $$\begin{aligned} \begin{aligned} dR_t&= \Big [\frac{\sigma ^2}{2R_t} - \mu R_t \Big ] dt + \sigma d\tilde{B}_t. \end{aligned} \end{aligned}$$

The second method is to consider $\bar{\mathbf Y}_t$ in polar coordinates with

$$\begin{aligned} d \bar{\mathbf Y}_t = A \bar{\mathbf Y}_t dt + \mathbf h_e dB_t, \end{aligned}$$

where $$\mathbf h_e=Q^{-1}\begin{pmatrix}0\\ \sigma _0\end{pmatrix} $$. Its norm $\bar{R}_t:= \Vert \bar{\mathbf Y}_t \Vert$ and its angle $\varvec{\theta }_t = \frac{\bar{\mathbf Y}_t}{\bar{R}_t}$ satisfy

$$\begin{aligned} d\bar{R}_t= & {} \Big [\frac{\Vert \mathbf h_e\Vert ^2-\langle \mathbf h_e,\varvec{\theta }_t\rangle ^2}{2 \bar{R}_t} - \mu \bar{R}_t \Big ] dt + \langle \varvec{\theta }_t,\mathbf h_e\rangle dB_t, \\ d\varvec{\theta }_t= & {} \Big [(A+\mu I) \varvec{\theta }_t - \frac{\Vert \mathbf h_e\Vert ^2 - \langle \mathbf h_e,\varvec{\theta }_t \rangle ^2}{2 \bar{R}_t^2} \varvec{\theta }_t\Big ] dt + \frac{1}{\bar{R}_t}\Big [ \mathbf h_e - \langle \mathbf h_e, \varvec{\theta }_t \rangle \varvec{\theta }_t\Big ]dB_t. \end{aligned}$$

By the averaging technique from (Baxendale and Greenwood 2011, Theorem 1) one can approximate $$\varvec{\theta }_t = \left( \begin{array}{c} \sin \nu t \\ \cos \nu t \\ \end{array} \right) $$, hence

4.4 $$\begin{aligned} \begin{aligned} d\bar{R}_t&=\left[ \frac{157.881 \sigma _0^2 - ( 1.27722 \sin \nu t + 12.5 \cos \nu t )^2 \sigma _0^2}{2 \bar{R}_t} - \mu \bar{R}_t \right] dt \\&\qquad + ( 1.27722 \sin \nu t + 12.5 \cos \nu t ) \sigma _0 dB_t. \end{aligned} \end{aligned}$$

**Fig. 5:**
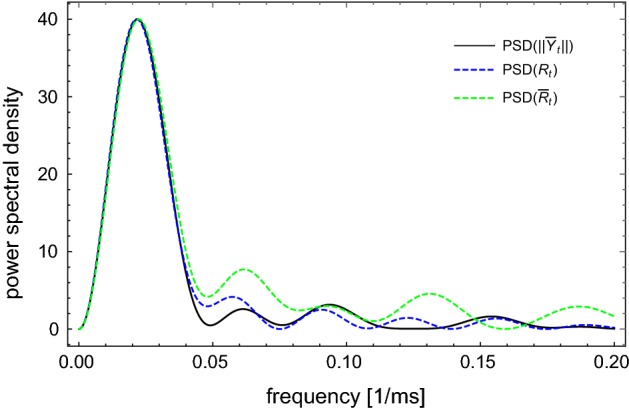
The norm of the process $\{\bar{\mathbf Y}_t\}$ (4.2), the process $\{R_t\}_t$ (4.3), and the process $\{\bar{R}_t\}_t$ (4.4) with the chosen parameters of the system, $\sigma _0=0.01$ and the same starting point $\Vert Q^{-1} \bar{\mathbf X}_0\Vert$ are compared by using the power spectral density. Their spectrum densities are well approximated (color figure online)

Thus, by using the averaging technique, we proved that both Eqs. (4.3) and (4.4) are good approximations of the radial process $\{\Vert \bar{\mathbf Y}_t\Vert \}_t = \{\Vert Q^{-1}\bar{\mathbf X}_t\Vert \}_t$. This can also be tested by using the power spectral density estimation (see Fig. 5).

### Firing mechanism

A spike in Eq. (3.1) occurs when there is a transition of a random trajectory from the vicinity of the stable fixed point $\mathbf X_e=(v_e,w_e)$ located on the left stable part of $\mathcal C_0$ to its right stable part and back to the vicinity of $\mathbf X_e$. This spike happens almost surely when a random trajectory with the starting point $\mathbf X_0$ in the vicinity of $\mathbf X_e$ crosses the threshold line $v=0$. From the phase space of Eq. (3.1) (see Fig. 2), the probability of a spike increases as the starting point $\mathbf X_0$ moves farther away from $\mathbf X_e$.

In order to construct the firing mechanism of Eq. (4.3) matching that of Eq. (3.1), we will calculate the conditional probability that Eq. (3.1) fires given that the trajectory crosses the line $L=\{(v_e,w): w \le w_e\}$. Denote by $L_i = (v_e,w_e-l_i)$ with $l_i = i\delta = i \frac{|w_e+0.453|}{20}$ for $i=0,1,\ldots ,34$, then the distance between the equilibrium and $L_i$ is $l_i$. The value $|w_e+0.453|$ can be considered as the distance between the fixed point $(v_e,w_e)$ and the separatrix (see also Fig. 1) along *L*. For a given pair ($\sigma _0, l_i$), a short trajectory starting in $L_i$ was simulated from (3.1), it was recorded whether a spike occurred (crossing the threshold $v=0$) in the first cycle of the stochastic path around $(v_e,w_e)$. This was repeated 1000 times and we counted the ratio of the number of spikes, denoted by $\hat{p}(l_i,\sigma _0)$, which is an estimate for the conditional probability of firing $p(l,\sigma _0)$. The estimation was, furthermore, repeated for $\sigma _0 = 0.001, 0.002, 0.003, 0.004, 0.005, 0.006, 0.007, 0.008, 0.009, 0.01, 0.015$.

From the numerical simulation, for each $\sigma _0$, the estimate of the conditional probability is close to zero when we start in the immediate neighborhood of the stable fixed point and close to one when we start at the $L_{34}$, i.e., sufficiently far from the fixed point. Theses estimates appear to depend in a sigmoidal way on the distance from the stable fixed point. Therefore we assumed the conditional probability of firing to be of the form

4.5 $$\begin{aligned} p(l)= \frac{1}{1+e^{\frac{a-l}{b}}}. \end{aligned}$$

**Fig. 6:**
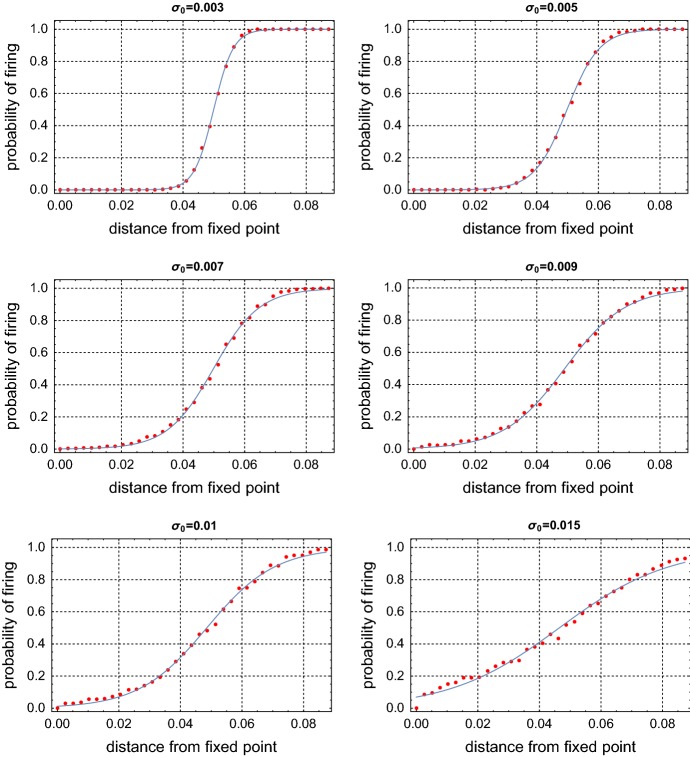
Conditional probability of spiking when crossing the line $L=\{(v_e,w): w \le w_e\}$ for different values of the noise amplitude $\sigma _0$. The red dots are individual nonparametric estimates and the blue curve are the fitted curves given by (4.5) (color figure online)

The parameters *a* and *b* then are estimated by using a non-linear regression from the above simulation data and are plotted in Fig. 6 for some different values of the noise amplitude $\sigma _0 = 0.003, 0.005, 0.007, 0.009, 0.01$, and 0.015. We see that the family of estimates, $\hat{p}$, fits the fitted curve quite well for each value of $\sigma _0$. Regression estimates are reported in Table 1. Note that $p(a)=1/2$, i.e., *a* is the distance along *L* from $w_e$ at which the conditional probability of firing equals one half. For all values of $\sigma _0$, the estimate of *a* is close to the distance along *L* between $w_e$ and the separatrix, which equals 0.05. In other words, the probability of firing, if the path starts at the intersection of *L* with the separatrix, is about 1 / 2. The estimate of *b* increases with respect to $\sigma _0$, and the conditional probability approaches a step function as the amplitude of the noise goes to zero. A step function would correspond to the firing being represented by a first passage time of a fixed threshold.

**Table 1:** Estimates of regression parameters for the conditional probability of firing in the original space (*a*, *b*) and in the transformed coordinates $(a^*, b^*)$ based on the additive noise $\sigma _0$

$\sigma _0$	0.001	0.002	0.003	0.004	0.005	0.006	0.007	0.008	0.009	0.01	0.015
*a*	0.050161	0.050268	0.049946	0.049760	0.049816	0.050001	0.049862	0.049411	0.049078	0.048559	0.046142
*b*	0.001028	0.002099	0.003192	0.004310	0.005281	0.006459	0.007478	0.008844	0.009877	0.011068	0.017722
$a^*$	0.630282	0.631624	0.627576	0.625240	0.625935	0.628262	0.626516	0.620859	0.616673	0.610148	0.579777
$b^*$	0.012918	0.026372	0.040106	0.054158	0.066352	0.081158	0.093960	0.111127	0.124107	0.139075	0.222676

To simplify calculations we will work on the transformed coordinates $\bar{\mathbf Y}_t$. Then the distance *l* between (0, *l*) and (0, 0) in $\bar{\mathbf X}_t$ transforms to the distance

$$\begin{aligned} r = \Bigg | Q^{-1} \begin{pmatrix}0 \\ l\end{pmatrix} \Bigg | = \sqrt{-\frac{m_{12}}{m_{21} \nu ^2}} l. \end{aligned}$$

and the conditional probability of firing Eq. (4.5) transforms to

4.6 $$\begin{aligned} p(r)= \frac{1}{1+e^{\frac{a^*-r}{b^*}}} \end{aligned}$$

where $$a^* = \sqrt{-\frac{m_{12}}{m_{21} \nu ^2}} a $$ and $$b^* = \sqrt{-\frac{m_{12}}{m_{21} \nu ^2}} b$$.

### ISI distributions

**Fig. 7:**
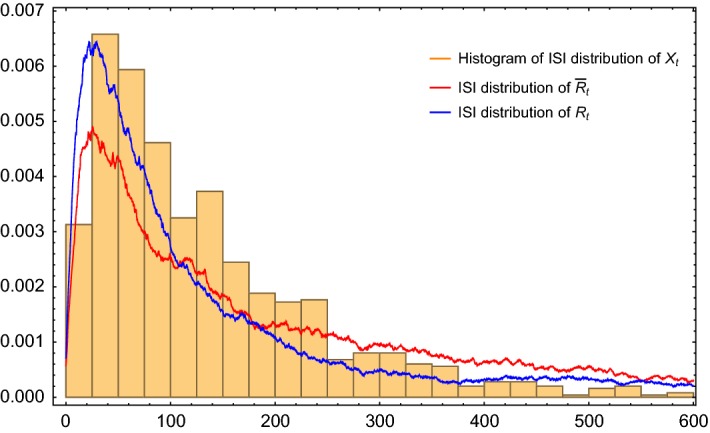
The estimated ISI distributions from our approximate LIF models (4.3) and (4.4) with the firing mechanism compare well with the estimated ISI histogram of FHN (3.1) reset to 0 after firings. $\sigma _0 = 0.01, M =1000, n=10$ (color figure online)

The comparison of the original stochastic FHN model (3.1) and the two LIF models (4.3) and (4.4) can be performed by studying the ISI statistics. Namely, one first simulates the trajectories of the system (3.1) with starting points $\mathbf X_0$ close to the fixed point $\mathbf X_e$ until the first spiking time, and thereafter resets to the starting points. Due to Theorem 3.2, we can simplify the simulation by choosing the starting point at exactly $\mathbf X_e$. This was done 1000 times, and the time of the first firing was recorded. A histogram for this data is shown in Fig. 7. The ISI-distribution of Eq. (4.3) is computed as follows (the ISI-distribution of Eq. (4.4) is computed similarly). Let $\tau _1$ be the first firing time. We computed the density of the distribution of $\tau _1$ in terms of the conditional hazard rate (Ditlevsen and Greenwood 2013),

$$\begin{aligned} \alpha (r,t) = \lim _{\Delta t \rightarrow 0} \frac{1}{\Delta t} P(t\le \tau _1 < t+\Delta t | \tau _1\ge t, R_{t} =r). \end{aligned}$$

This function is the density of the conditional probability, given the position on *L* is *r* at time *t*, of a spike occurring in the next small time interval, given that it has not yet occurred.

Notice that the estimated conditional probability of firing (4.6) is calculated in one cycle of the process, which on average takes $2\pi /\nu$ time units. Therefore, we estimate the hazard rate as

4.7 $$\begin{aligned} \alpha (r,t) = \alpha (r) = \frac{\nu }{2 \pi } \frac{1}{1+e^{\frac{a^*-r}{b^*}}} . \end{aligned}$$

On the other hand, from standard results from survival analysis, see e.g. Aalen and Borgan (2008) we know that the density of the firing time can be calculated as

4.8 $$\begin{aligned} g(t) = \frac{d}{dt} P(\tau _1 \le t) = E \Bigg ( \alpha (R_t) e^{-\int _0^t \alpha (R_s)ds}\Bigg ). \end{aligned}$$

Due to the law of large numbers, for fixed *t*, we can numerically determine the density (4.8) up to any desired precision by choosing *n* and *M* large enough through the expression

$$\begin{aligned} g(t) \approx \frac{1}{M} \sum \limits _{m=1}^M \alpha (R^{(m)}_t) e^{-\frac{t}{n} \sum \limits _{i=1}^n \frac{\alpha \big (R^{(m)}_{it/n}\big )+\alpha \big (R^{(m)}_{(i-1)t/n}\big )}{2}}. \end{aligned}$$

Here ($R^{(m)}_0, \ldots , R^{(m)}_{it/n}, \ldots , R^{(m)}_{t})$ are *M* realizations of $R_{it/n}, i = 0,1,\ldots , n$, and the integral has been approximated by the trapezoidal rule. The results are illustrated in Fig. 7 for $\sigma _0=0.01$, using $M = 1000, n=10$. The estimated ISI distributions from our approximate LIF models (4.3) and (4.4) with the firing mechanism compare well with the estimated ISI histogram of FHN (3.1) reset to 0 after firings.

## Appendix

### Proof of Theorem 3.1

We are going to prove that there exists a random pullback attractor for the general Eq. (3.2). Consider two cases:

- Additive noise: In this case, the proof follows similar steps as in Garrido-Atienza et al. (2009). We define $\mathbf Y_t = \mathbf X_t - \eta _t$ where $\eta _t$ is the unique stationary solution of
  $$\begin{aligned} d\eta _t = -\eta _t dt + (0,\sigma _0)^T dB_t. \end{aligned}$$
  System (3.2) is then tranformed to
  5.1 $$\begin{aligned} \dot{\mathbf Y}_t = F(\mathbf Y_t + \eta _t) + \eta _t. \end{aligned}$$
  Observe that
  $$\begin{aligned} \frac{d}{dt} \Vert \mathbf Y_t\Vert ^2= & {} 2 \langle \mathbf Y_t, F(\mathbf Y_t + \eta _t) - F(\eta _t)\rangle + 2 \langle \mathbf Y_t, F(\eta _t) + \eta _t \rangle \\\le & {} 2(a-b\Vert \mathbf Y_t\Vert ^2) + b\Vert \mathbf Y_t\Vert ^2 + \frac{1}{b} \Vert F(\eta _t) + \eta _t\Vert ^2 \\= & {} 2a + \frac{1}{b} \Vert F(\eta _t) + \eta _t\Vert ^2 - b \Vert \mathbf Y_t\Vert ^2. \end{aligned}$$
  Hence by the comparison principle, $\Vert \mathbf Y_t\Vert \le R_t$ whenever $\Vert \mathbf Y_0\Vert ^2 \le R_0$ where $R_t$ is the solution of
  5.2 $$\begin{aligned} \dot{R}_t = 2a + \frac{1}{b} \Vert F(\eta _t) + \eta _t\Vert ^2 - b R_t, \end{aligned}$$
  which can be computed explicitly as
  $$\begin{aligned} R_t(\omega ,R_0) = e^{-bt}R_0 + \int _0^t e^{-b(t-s)} \left[ 2a + \frac{1}{b} \Vert F(\eta _s) + \eta _s\Vert ^2 \right] ds. \end{aligned}$$
  It is then easy to check that the vector field in (5.1) satisfies the local Lipschitz property and the solution is bounded and thus of linear growth on any fixed [0, *T*], see e.g. Schenk-Hoppé (1996). Hence there exists a unique solution of (5.1) with initial condition, which also proves the existence and uniqueness of the solution of (3.2). The cocycle property (3.7) follows automatically from (Arnold 1998, Chapter 2). A direct computation shows that there exists a random radius
  $$\begin{aligned} R^*(\omega ) = \int _{-\infty }^{0} \left[ 2a + \frac{1}{b} \Vert F(\eta _s) + \eta _s\Vert ^2 \right] e^{bs} ds, \end{aligned}$$
  which is the stationary solution of (5.2), such that $\mathbf X_t(\omega ,\mathbf X_0) \in B(\eta _t,R^*(\theta _t \omega ))$ whenever $\mathbf X_0 \in B(\eta _0,R^*(\omega ))$ by the comparison principle, and furthermore,
  $$\begin{aligned} \limsup \limits _{t\rightarrow \infty }\Vert \mathbf Y_t(\theta _{-t}\omega ,\mathbf Y_0)\Vert ^2 \le \limsup \limits _{t \rightarrow \infty } R_t(\theta _{-t} \omega ,R_0) = R^*(\omega ). \end{aligned}$$
  Hence the random ball $B(\eta ,R^*)$ is a forward invariant pullback absorbing set of the random dynamical system generated by $\varphi (t,\omega )\mathbf X_0$ (3.2). By the classical theorem Crauel et al. (1997), there exists the global random pullback attractor for (3.2).
- Multiplicative noise: In this case, we introduce the transformation
  5.3 $$\begin{aligned} \mathbf Y_t = (v_t,\bar{\omega }_t)^T:= \begin{pmatrix} 1&{}\,0\\ 0&{}\,e^{-\sigma _0 z_t} \end{pmatrix}\mathbf X_t = T(z_t)\mathbf X_t \end{aligned}$$
  where $z_t$ is the unique stationary solution of the Ornstein–Uhlenbeck equation
  5.4 $$\begin{aligned} d z_t = - z_t dt + dB_t. \end{aligned}$$
  This transforms system (3.2) into a random differential equation.
  5.5 $$\begin{aligned} \dot{v_t}= & {} v_t -\frac{v_t^3}{3} - e^{\sigma _0 z_t} \bar{\omega }_t + I \nonumber \\ \dot{\bar{\omega }}_t= & {} e^{-\sigma _0 z_t} \varepsilon v_t + (\sigma _0 z_t - \varepsilon \beta ) \bar{\omega }_t + \varepsilon \alpha e^{-\sigma _0 z_t}. \end{aligned}$$
  or equivalently,
  $$\begin{aligned} \dot{\mathbf Y}_t = G(z_t,\mathbf Y_t) \end{aligned}$$
  where *G* satisfies $G(z_t,0) = (I,\varepsilon \alpha e^{-\sigma _0 z_t})^T$ and
  $$\begin{aligned}&\langle \mathbf Y_1 - \mathbf Y_2,G(z_t,\mathbf Y_1)-G(z_t,\mathbf Y_2) \rangle \\&\quad = (v_1-v_2)^2 \left[ 1- \frac{1}{3} \left( v_1^2 + v_1 v_2 + v_2^2\right) \right] \\&\quad \quad +(\epsilon e^{-\sigma _0 z_t} - e^{\sigma _0 z_t})(v_1-v_2)(\bar{w}_1-\bar{w}_2)\\&\quad \quad +(\sigma _0 z_t -\epsilon \beta ) (\bar{w}_1-\bar{w}_2)^2\\&\quad \le (v_1-v_2)^2 - \frac{1}{12}(v_1-v_2)^4 + \frac{1}{2\epsilon \beta } \left( \epsilon e^{-\sigma _0 z_t} - e^{\sigma _0 z_t}\right) ^2 (v_1-v_2)^2\\&\quad \quad + \left( \sigma _0 z_t -\frac{\epsilon \beta }{2}\right) (\bar{w}_1-\bar{w}_2)^2\\&\quad \le - \frac{1}{12}(v_1-v_2)^4 + \left[ 1+ \frac{1}{2\epsilon \beta } \left( \epsilon e^{-\sigma _0 z_t} - e^{\sigma _0 z_t}\right) ^2\right. \\&\left. \quad \quad - \sigma _0 z_t +\frac{\epsilon \beta }{2}\right] (v_1-v_2)^2 \\&\quad \quad + \left( \sigma _0 z_t -\frac{\epsilon \beta }{2}\right) \Vert \mathbf Y_1-\mathbf Y_2\Vert ^2\\&\quad \le -\frac{1}{12} \Big ((v_1-v_2)^2 + 6 \left[ 1+ \frac{1}{2\epsilon \beta } \left( \epsilon e^{-\sigma _0 z_t} - e^{\sigma _0 z_t}\right) ^2 \right. \\&\left. \quad \quad - \sigma _0 z_t +\frac{\epsilon \beta }{2}\right] \Big )^2 \\&\quad \quad + 3 \left[ 1+ \frac{1}{2\epsilon \beta } \left( \epsilon e^{-\sigma _0 z_t} - e^{\sigma _0 z_t}\right) ^2 - \sigma _0 z_t +\frac{\epsilon \beta }{2}\right] ^2 \\&\quad \quad + \Big (\sigma _0 z_t -\frac{\epsilon \beta }{2}\Big ) \Vert \mathbf Y_1-\mathbf Y_2\Vert ^2\\&\quad \le 3 \left[ 1+ \frac{1}{2\epsilon \beta } \left( \epsilon e^{-\sigma _0 z_t} - e^{\sigma _0 z_t}\right) ^2 - \sigma _0 z_t +\frac{\epsilon \beta }{2}\right] ^2 \\&\quad \quad + \Big (\sigma _0 z_t -\frac{\epsilon \beta }{2}\Big ) \Vert \mathbf Y_1-\mathbf Y_2\Vert ^2. \end{aligned}$$
  Thus,
  $$\begin{aligned} \frac{d}{dt} \Vert \mathbf Y_t\Vert ^2= & {} 2 \langle \mathbf Y_t-0, G(z_t, \mathbf Y_t) - G(z_t,0)\rangle + 2 \langle \mathbf Y_t, G(z_t,0) \rangle \\\le & {} 3 \left[ 1+ \frac{1}{2\epsilon \beta } \left( \epsilon e^{-\sigma _0 z_t} - e^{\sigma _0 z_t}\right) ^2 - \sigma _0 z_t +\frac{\epsilon \beta }{2} \right] ^2 \\&+\Big (\sigma _0 z_t -\frac{\epsilon \beta }{2}\Big ) \Vert \mathbf Y_t\Vert ^2 + 2 \langle \mathbf Y_t, G(z_t,0) \rangle \\\le & {} 3 \left[ 1+ \frac{1}{2\epsilon \beta } \left( \epsilon e^{-\sigma _0 z_t} - e^{\sigma _0 z_t}\right) ^2 - \sigma _0 z_t +\frac{\epsilon \beta }{2} \right] ^2 \\&+ \frac{4}{\varepsilon \beta } \Vert G(z_t,0)\Vert ^2 + \Big (\sigma _0 z_t -\frac{\epsilon \beta }{4}\Big ) \Vert \mathbf Y_t\Vert ^2 \\\le & {} 3 \left[ 1+ \frac{1}{2\epsilon \beta } \left( \epsilon e^{-\sigma _0 z_t} - e^{\sigma _0 z_t}\right) ^2 - \sigma _0 z_t +\frac{\epsilon \beta }{2} \right] ^2 \\&+ \frac{4}{\varepsilon \beta } \Big [I^2 + \varepsilon ^2 \alpha ^2 e^{-2 \sigma _0 z_t}\Big ]+(\sigma _0 z_t -\frac{\epsilon \beta }{4}) \Vert \mathbf Y_t\Vert ^2 \\\le & {} p(z_t) + q(z_t) \Vert \mathbf Y_t\Vert ^2. \end{aligned}$$
  Hence by the comparison principle, $\Vert \mathbf Y_t\Vert ^2 \le R_t$ whenever $\Vert \mathbf Y_0\Vert ^2 \le R_0$ where $R_t$ is the solution of
  5.6 $$\begin{aligned} \dot{R}_t = p(z_t) + q(z_t) R_t, \end{aligned}$$
  which can be computed explicitly as
  $$\begin{aligned} R_t(\omega ,R_0) = e^{\int _0^t q(z_u(\omega )) du} R_0 + \int _{0}^{t} p(z_s(\omega )) e^{\int _s^t q(z_u(\omega )) du} ds. \end{aligned}$$
  Using similar arguments as in the additive noise case, there exists a unique solution of (5.5) and (3.2). Also, the solution generates a random dynamical system. On the other hand, observe that by the Birkhorff ergodic theorem, there exists almost surely
  $$\begin{aligned} \lim \limits _{t \rightarrow -\infty }\frac{1}{t} \int _t^0 q(z_u) du = \lim \limits _{t\rightarrow -\infty }\frac{1}{t} \int _t^0 q(z(\theta _u \omega )) = E \Big [\sigma _0 z(\cdot ) - \frac{\varepsilon \beta }{4}\Big ] = -\frac{\varepsilon \beta }{4} <0, \end{aligned}$$
  therefore there exists a unique stationary solution of (5.6) which can be written in the form
  $$\begin{aligned} \bar{R}(\omega ) = \int _{-\infty }^{0} p(z_s(\omega )) e^{\int _s^0 q(z_u(\omega )) du} ds. \end{aligned}$$
  Moreover, $\Vert \mathbf Y_t(\omega ,\mathbf Y_0)\Vert ^2 \le \bar{R}(\theta _t \omega )$ whenever $\Vert \mathbf Y_0\Vert ^2 \le \bar{R}(\omega )$ and
  $$\begin{aligned} \limsup \limits _{t\rightarrow \infty }\Vert \mathbf Y_t(\theta _{-t}\omega ,\mathbf Y_0)\Vert ^2 \le \limsup \limits _{t \rightarrow \infty } R_t(\theta _{-t} \omega ,R_0) = \bar{R}(\omega ). \end{aligned}$$
  Hence, the ball $B(0,R(\omega ))$ is actually forward invariant under the random dynamical system generated by (5.5) and is also a pullback absorbing set. Again by applying Crauel et al. (1997), there exists a random attractor for (5.5). Due to the fact that $z_t$ is the stationary solution of (5.4), it is easy to see that the random linear transformation *T*(*z*) given in (5.3) is *tempered* (see Arnold 1998, pp. 164, 386), i.e.
  $$\begin{aligned} 0\le \lim \limits _{t \rightarrow \infty } \frac{1}{t} \log \Vert T(z_t)\Vert = \lim \limits _{t \rightarrow \infty } \frac{1}{2t} \log (1+ e^{-2\sigma _0 z_t}) \le \lim \limits _{t \rightarrow \infty } \frac{1}{2t} (1 + 2\sigma _0 |z_t|) = 0. \end{aligned}$$
  Therefore, it follows from Imkeller and Schmalfuss (2001) that systems (3.2) and (5.5) are conjugate under the tempered transformation (5.3), hence there exists also a random attractor for system (3.2).

$$\square $$

### Proof of Theorem 3.2

Observe that the matrix

$$\begin{aligned} DF(\mathbf X_e) = \begin{pmatrix} m_{11}&{}\,m_{12}\\ m_{21}&{}\, m_{22} \end{pmatrix} \end{aligned}$$

has two conjugate complex eigenvalues with negative real part

$$\begin{aligned} \lambda _{1,2}= & {} \frac{1}{2}\left( 1-v_e^2 - \epsilon \beta \right) \pm \frac{i}{2} \sqrt{4\epsilon - (1-v_e^2+\epsilon \beta )^2}\\= & {} -0.0730077\pm 0.31615 i = -\mu \pm \nu i. \end{aligned}$$

Hence by using the transformation $\mathbf X-\mathbf X_e=Q \mathbf Y$ and $\bar{X} = Q \bar{\mathbf Y}$ with

$$\begin{aligned} Q=\begin{pmatrix} -\nu &{}\,m_{11}+\mu \\ 0&{}\, m_{21} \end{pmatrix}, \end{aligned}$$

the equations (3.8) and (3.9) are transformed into the normal forms

5.7 $$\begin{aligned} d\mathbf Y_t= & {} \Big [Q^{-1}DF(\mathbf X_e)Q \mathbf Y_t + Q^{-1}\bar{F}(Q \mathbf Y_t) \Big ] dt \nonumber \\&+ Q^{-1}H(Q\mathbf Y_t+ \mathbf X_e) \circ dB_t \end{aligned}$$

5.8 $$\begin{aligned}= & {} [A \mathbf Y_t + F_1(\mathbf Y_t)] dt + Q^{-1}H(Q\mathbf Y_t+ \mathbf X_e) \circ dB_t, \nonumber \\ \mathbf Y_0= & {} Q^{-1}(\mathbf X_0-\mathbf X_e), \end{aligned}$$

and

5.9 $$\begin{aligned} d{\bar{\mathbf Y}}_t= & {} A \bar{\mathbf Y}_t dt + Q^{-1}H(Q\bar{\mathbf Y}_t+\mathbf X_e) \circ dB_t, \nonumber \\ \bar{\mathbf Y}_0= & {} Q^{-1}(\mathbf X_0 -\mathbf X_e). \end{aligned}$$

where

$$\begin{aligned} A = Q^{-1} DF(\mathbf X_e) Q = \begin{pmatrix}-\mu &{} \nu \\ -\nu &{} -\mu \end{pmatrix};\qquad F_1(\mathbf Y) :=Q^{-1}\bar{F}(Q\mathbf Y), \end{aligned}$$

and

5.10 $$\begin{aligned} \Vert F_1(\mathbf Y)\Vert \le \gamma (r) \Vert Q^{-1}\Vert \Vert Q\mathbf Y\Vert \le \Vert Q^{-1}\Vert \gamma (r)r,\qquad \forall \Vert \mathbf Y\Vert \le \frac{r}{\Vert Q\Vert }. \end{aligned}$$

Define the difference $\mathbf Z_t := \mathbf Y_t - \bar{\mathbf Y}_t$, then $\mathbf Z_t$ satisfies

$$\begin{aligned} d\mathbf Z_t= & {} [A \mathbf Z_t + F_1(\mathbf Y_t)]dt + B_1 \mathbf Z_t \circ dB_t\\= & {} \Big [(A+\frac{1}{2}B_1^TB_1) \mathbf Z_t + F_1(\mathbf Y_t)\Big ]dt + B_1 \mathbf Z_t dB_t, \end{aligned}$$

where

$$\begin{aligned} B_1 := 0 \text { if } H(\mathbf X) = (0,\sigma _0)^T\quad \text {and}\quad B_1 := Q^{-1}BQ \text { if } H(\mathbf X) = B\mathbf X. \end{aligned}$$

We analyze these two cases separately.

- Additive noise: then the equation for $\mathbf Z_t$ becomes deterministic, hence
  $$\begin{aligned} \frac{d}{dt}\Vert \mathbf Z_{t\wedge \tau }\Vert ^2= & {} 2 \Big \langle \mathbf Z_{t\wedge \tau }, A \mathbf Z_{t\wedge \tau } + F_1(\mathbf Y_{t\wedge \tau })\Big \rangle \\\le & {} - 2 \mu \Vert \mathbf Z_{t\wedge \tau }\Vert ^2 + \mu \Vert \mathbf Z_{t\wedge \tau }\Vert ^2 + \frac{1}{\mu } \Vert F_1(\mathbf Y_{t\wedge \tau })\Vert ^2 \\\le & {} \frac{1}{\mu } \Vert Q^{-1}\Vert ^2 \gamma (r)^2 r^2 - \mu \Vert \mathbf Z_{t\wedge \tau }\Vert ^2. \end{aligned}$$
  Using the fact that $\mathbf Z_0 = 0$, it follows that
  $$\begin{aligned} \Vert \mathbf Z_{t\wedge \tau }\Vert ^2 \le \frac{1}{\mu ^2} \Vert Q^{-1}\Vert ^2 \gamma (r)^2 r^2 + e^{-\mu (t\wedge \tau )} \Big (\Vert \mathbf Z_0\Vert ^2 - \frac{1}{\mu ^2} \Vert Q^{-1}\Vert ^2 \gamma (r)^2 r^2 \Big ). \end{aligned}$$
  Therefore,
  $$\begin{aligned} \sup _{t \le \tau } \Vert \mathbf Z_t\Vert \le \frac{1}{\mu } \Vert Q^{-1}\Vert \gamma (r) r \end{aligned}$$
  which proves (3.10) with $C = \frac{1}{\mu } \Vert Q\Vert \Vert Q^{-1}\Vert$.
- Multiplicative noise: By Ito’s formula for the stopping time,
  $$\begin{aligned} d\Vert \mathbf Z_{t\wedge \tau }\Vert ^2= & {} 2 \Big \langle \mathbf Z_{t\wedge \tau }, (A+\frac{1}{2}B_1^TB_1) \mathbf Z_{t\wedge \tau } + F_1(\mathbf Y_{t\wedge \tau })\Big \rangle d(t\wedge \tau ) \\&+ \Vert B_1 \mathbf Z_{t\wedge \tau }\Vert ^2 d({t\wedge \tau }) \\&+ 2 \langle \mathbf Z_{t\wedge \tau },B_1 \mathbf Z_{t\wedge \tau }\rangle d B_{t\wedge \tau }, \end{aligned}$$
  hence taking the expectation on both sides and using (5.10) we have
  $$\begin{aligned} \frac{d}{dt}E\Vert \mathbf Z_{t\wedge \tau }\Vert ^2\le & {} 2\Big (-\mu + \Vert B_1^TB_1\Vert \Big )E\Vert \mathbf Z_{t\wedge \tau }\Vert ^2 + 2 \Vert Q^{-1}\Vert \gamma (r)r E \Vert \mathbf Z_{t\wedge \tau }\Vert \\\le & {} (-\mu + 2 \Vert B_1^TB_1\Vert )E\Vert \mathbf Z_{t\wedge \tau }\Vert ^2 \\&+ \Big [-\mu \big (E\Vert \mathbf Z_{t\wedge \tau }\Vert \big )^2 +2 \Vert Q^{-1}\Vert \gamma (r)r E \Vert \mathbf Z_{t\wedge \tau }\Vert \Big ] \\\le & {} (-\mu + 2 \Vert B_1^TB_1\Vert )E\Vert \mathbf Z_{t\wedge \tau }\Vert ^2 + \frac{1}{\mu } \Vert Q^{-1}\Vert ^2\gamma (r)^2r^2, \end{aligned}$$
  where the last inequality follows from the Cauchy inequality. Since
  5.11 $$\begin{aligned} \lambda = \mu - 2 \Vert B^T_1 B_1\Vert >0, \end{aligned}$$
  by noting that $\mathbf Z_0 = 0$, we get
  $$\begin{aligned} E\Vert \mathbf Z_{t\wedge \tau }\Vert ^2\le & {} E\Vert \mathbf Z_{0\wedge \tau }\Vert ^2 e^{-\lambda (t\wedge \tau )}+ \frac{1}{\mu } \Vert Q^{-1}\Vert ^2\gamma (r)^2r^2 \ \frac{1}{\lambda } \Big [1 - e^{-\lambda (t\wedge \tau )}\Big ]\\\le & {} \frac{1}{\mu } \frac{1}{\lambda }\Vert Q^{-1}\Vert ^2\gamma (r)^2r^2, \end{aligned}$$
  which proves (3.11) by choosing $C := \frac{1}{\mu } \frac{1}{\lambda }\Vert Q^{-1}\Vert ^2 \Vert Q\Vert ^2$.

$$\square $$
